# Phylogenetic Analyses and Morphological Studies Reveal Four New Species of *Phellodon* (Bankeraceae, Thelephorales) from China

**DOI:** 10.3390/jof9010030

**Published:** 2022-12-23

**Authors:** Chang-Ge Song, Yi-Fei Sun, Shun Liu, Yuan-Yuan Chen, Bao-Kai Cui

**Affiliations:** 1 Institute of Microbiology, School of Ecology and Nature Conservation, Beijing Forestry University, Beijing 100083, China; 2 College of Forestry, Henan Agricultural University, Zhengzhou 450002, China

**Keywords:** Basidiomycota, ectomycorrhizal fungi, macrofungi, new species, stipitate hydnoid fungi

## Abstract

*Phellodon* is a genus of ectomycorrhizal fungi with important ecological roles and exploitable biological activities. In this study, four new species of *Phellodon*, *P. caesius*, *P. henanensis*, *P. concentricus* and *P. subgriseofuscus*, are described from China based on morphological characters and molecular evidence. The phylogenetic analyses of *Phellodon* were carried out based on the ITS + nLSU gene regions and the combined sequence dataset of ITS + nLSU + nSSU + RPB1 + RPB2 gene regions. *Phellodon caesius* is characterized by its dark bluish-grey, dark grey to black grey pileus, ash grey to dark bluish-grey spines, and the presence of both simple septa and clamp connections on generative hyphae of stipe. *Phellodon concentricus* is characterized by its zonate pileal surface, dark grey context in pileus, and spongy basidiomata. *Phellodon henanensis* is characterized by its ash grey, light vinaceous grey to light brown pileal surface, thin context in pileus, and the presence of both simple septa and clamp connections on generative hyphae of spines. *Phellodon subgriseofuscus* is characterized by its fuscous to black pileal surface, white to light brown spines, and vinaceous grey context. Illustrated descriptions and the ecological habits of the novel species are provided.

## 1. Introduction

*Phellodon* P. Karst., a genus of Bankeraceae, is a kind of stipitate hydnoid fungi. It was established by Petter Adolf Karsten in 1881 and typified by *P. niger* (Fr.) P. Karst [1]. Species in *Phellodon* are characterized by basidiome pileate and stipitate; pileus white to yellow-brown or grey-brown in various hues or olivaceous to black; basidia clavate, 4-spored, without basal clamp; spores broadly ellipsoid to subglobose, spinulose; cystidia lacking; odor of fenugreek when dried [2].

Species in *Phellodon* are a group of ectomycorrhizal fungi with important ecological roles [3]. The symbiotic relationship between mycorrhizal and host plants plays an essential role in nutrient cycling, energy flow, community species composition, biodiversity, and ecosystem change in forest ecosystems [4]. As significant ectomycorrhizal fungi, stipitate hydnoid fungi connected with plant roots can reflect the conservation state of forest ecosystems [5]. They can promote the absorption of nutrients by plants, which in turn promotes the circulation of materials in the ecosystem [3]. In addition, some species in *Phellodon* have exploitable biological activity. Stadler and Anke [6] conducted a study on *Phellodon melaleucus* (Sw. ex Fr.) P. Karst. and isolated a new antibiotic Phellodonic Acid from it. Reekie et al. [7] isolated a biologically active and highly functionalized hirsute derivative from the Tasmanian fungus *Phellodon melaleucus* and proposed the chemoenzymatic total synthesis of phellodonic acid. Fang et al. [8] isolated cyathane diterpenoids and nitrogenous terphenyl derivative from the fruiting bodies of basidiomycete *Phellodon niger*. Therefore, taxonomic and phylogenetic studies on *Phellodon* can lay the foundation for exploring their ecological functions and biological activities.

Fries originally placed the species of *Phellodon* in his tribe Mesopus, section Lignosa, which was made to include all tough mesopodous species of the Hydnaceae [9]. At that time, species of *Phellodon* were considered members of Hydnaceae. In 1961, Donk established Bankeraceae and made *Bankera* Coker and Beers and *Phellodon* members of the family [10]. Baird et al. [11] recombined *Bankera fuligineoalba* (J.C. Schmidt) Pouzar, the typified species of *Bankera*, to *Phellodon*. Since then, *Bankera* has been incorporated into *Phellodon*. From 1956 to 2005, morphological characteristics of *Phellodon* were systematically and deeply studied in North America and Europe [2,12,13,14,15,16,17,18,19,20,21,22]. Subsequently, with the development of molecular systematics, DNA sequence analysis was gradually introduced into the taxonomic and phylogenetic studies of the Bankeraceae [11,23,24,25]. However, these studies only focus on the internal transcribed Spacer (ITS) sequences, and there are still many unanswered questions. Baird et al. [11] reevaluated the species of stipitate hydnums from the southern United States and identified 41 distinct taxa of *Hydnellum*, *Phellodon*, and *Sarcodon*. They conducted a phylogenetic study based on ITS sequence and proved that *Phellodon* is independent of *Hydnellum* and *Sarcodon*. Li [26] conducted a systematic study of the Bankeraceae in Korea using ITS, the large subunit of nuclear ribosomal RNA gene (nLSU), and the second largest subunit of RNA polymerase II (RPB2) sequences, and 17 species were determined including the genus *Phellodon*. It was the first analysis of the family based on multigene sequences, but the number of species included in this phylogenetic analysis is relatively limited because many species do not have available sequences. In recent years, taxonomic and phylogenetic studies of *Phellodon* have been carried out in China, and multiple gene fragments of *Phellodon* have been provided. Song et al. [27] described four new species of *Phellodon* from southern China and provided the available sequences of nLSU, the small subunit of nuclear ribosomal RNA gene (nSSU), the small subunit of mitochondrial rRNA gene (mtSSU), the largest subunit of RNA polymerase II (RPB1), and RPB2 genes of *Phellodon*. Phylogenetic trees were constructed based on the combined ITS + nLSU + nSSU + RPB1 + RPB2 sequences, which confirmed the affinities of three new species and reveal the relationships of *Phellodon* species [28]. About 33 species have been described and transferred to the genus according to Index Fungorum (http://www.indexfungorum.org/ (accessed on 26 April 2022)). So far, eight species of *Phellodon* have been described in China [27,28,29], which means that the genus may have a relatively large distribution in China.

Macrofungi have important ecological and economical values. The species diversity, taxonomy, and phylogeny of macrofungi have been extensively investigated in recent years, and many new species have been discovered [30,31,32,33,34,35,36,37,38,39,40,41,42,43,44,45,46,47]. During our investigations of macrofungi in China, numerous specimens of *Phellodon* were collected. In the current study, the phylogenetic analyses of *Phellodon* were carried out based on the ITS + nLSU gene regions and the combined sequence dataset of ITS + nLSU + nSSU + RPB1 + RPB2 gene regions. Subsequent morphological and molecular studies uncovered four undescribed species. These species are described and illustrated below.

## 2. Materials and Methods

### 2.1. Morphological Studies

The specimens used in this study were collected during the annual growing season of macrofungi. At the same time, the specimen information, host trees, ecological habits, location, altitude, collector, and date were recorded, and photos of the fruiting bodies and growth environment were taken. The location information and ecological habits of the specimens mentioned above are stated in the results section. All samples examined in this study were deposited at the herbaria of the Institute of Microbiology, Beijing Forestry University, China (BJFC). Micro-morphological data were obtained from dried specimens and observed under a light microscope (Nikon Eclipse E 80i microscope, Nikon, Tokyo, Japan) following methods in Liu et al. [38].

Samples for microscopic examination were mounted in Cotton Blue, Melzer and 5% potassium hydroxide (KOH), separately. Basidiospores were measured from sections cut from the spines. The following abbreviations are used: IKI, Melzer’s reagent; IKI–, neither amyloid nor dextrinoid; KOH, 5% potassium hydroxide; CB, Cotton Blue; CB–, acyanophilous; *L* = mean spore length, *W* = mean spore width, *Q* = L/W ratio, *n* (a/b) = number of spores (a) measured from given number of specimens (b). A field Emission Scanning Electron Microscope (FESEM) Hitachi SU-8010 (Hitachi, Ltd., Tokyo, Japan) was used to film the spore’s morphology, and the materials were studied at up to 1800 times magnification, according to the method by Sun et al. [45].

### 2.2. DNA Extraction, PCR Amplification, and Sequencing

A CTAB plant genome rapid extraction kit-DN14 (Aidlab Biotechnologies Co., Ltd.) was employed for DNA extraction from dried specimens. The extracted DNA were used to perform the polymerase chain reaction (PCR) according to the manufacturer’s instructions with some modifications [34,40]. The primer pairs ITS5/ITS4, LR0R/LR7, NS1/NS4, AF/Cr, and 5F/7Cr were used to amplify ITS, nLSU, nSSU, RPB1, and RPB2 sequences [27,28]. The concentration of all primers is 1 g per mL. The final Polymerase Chain Reaction (PCR) volume was 30 μL; each tube contained 1 μL each primer, 1 μL extracted DNA, 12 μL ddH2O, and 15 μL 2 × EasyTaq PCR Supermix (TransGen Biotech Co., Ltd., Beijing, China). PCRs were performed on S1000™ Thermal Cycler (Bio-Rad Laboratories, California, USA). The PCR procedure for ITS was: initial denaturation at 95 °C for 3 min, followed by 34 cycles of denaturation at 94 °C for 40 s, annealing at 56 °C for 45 s and extension at 72 °C for 1 min, and a final extension at 72 °C for 10 min. The PCR process for nLSU and nSSU was as follows: initial denaturation at 94 °C for 1 min, followed by 35 cycles at 94 °C for 30 s, 50 °C for 1 min, 72 °C for 90 s, and a final extension of 72 °C for 10 min. The PCR process for RPB1 and RPB2 was as follows: initial denaturation at 94 °C for 2 min, 9 cycles at 94 °C for 45 s, 60 °C for 45 s, followed by 36 cycles at 94 °C for 45 s, 53 °C for 1 min, 72 °C for 90 s and a final extension of 72 °C for 10 min. The PCR products were purified and sequenced at the Beijing Genomics Institute, China, with the same primers. All sequences analyzed in this study were deposited at GenBank and listed in Table 1.

### 2.3. Phylogenetic Analyses

The phylogenetic relationships of *Phellodon* were analyzed by the datasets of combined ITS + nLSU sequences and ITS + nLSU + nSSU + RPB1 + RPB2 sequences. The ITS + nLSU sequences were used to infer the phylogeny of *Phellodon*. The 5-gene datasets more specifically showed the differences between *Phellodon* species. The sequences generated in this study and retrieved from GenBank were combined with ITS, nLSU, nSSU, RPB1, and RPB2 sequences of *Phellodon* and outgroups. *Amaurodon aquicoeruleus* Agerer (UK 452) and *A. viridis* (Alb. and Schwein.) J. Schröt (TAA 149664) were used as the outgroups, according to Song et al. [28]. The datasets were aligned in MAFFT 7 [46] and manually adjusted in BioEdit [47]. Alignments were spliced in Mesquite v. 3.2. [48]. The congruences of the 5-gene (ITS, nLSU, nSSU, RPB1, and RPB2,) were evaluated with the incongruence length difference (ILD) test [49] implemented in PAUP* version 4.0b10 [50], under heuristic search and 1000 homogeneity replicates. The best-fit evolutionary model was selected with AIC (Akaike Information Criterion) using jModelTest for each partition [51,52]. Phylogenetic analyses were carried out according to previous studies [31,40].

Maximum parsimony (MP) analysis was performed in PAUP*version 4.0b10 [50] with the heuristic search. All characters were equally weighted and gaps were treated as missing data. Trees were inferred using the heuristic search option with TBR branch swapping and 1000 random sequence additions. Max-trees was set to 5000, branches of zero length were collapsed and all parsimonious trees were saved. Clade robustness was assessed using a bootstrap analysis with 1000 replicates [53]. Descriptive tree statistics, tree length (TL), consistency index (CI), retention index (RI), rescaled consistency index (RC), and homoplasy index (HI) were calculated for each Maximum Parsimonious Tree (MPT) generated. Only the Maximum Parsimony best tree from all searches was kept. Maximum Likelihood (ML) analysis was performed in RAxmL v.7.2.8 with a GTR + G + I model [54]. All model parameters were estimated by the program, but only the best maximum likelihood tree from all searches was kept. MrModeltest 2.3 [55,56] was used to determine the best-fit evolution model for each dataset for Bayesian inference (BI). BI was performed using MrBayes 3.2.6 on Abe through the Cipres Science Gateway (www.phylo.org, accessed on 23 April 2022) with 2 independent runs, each one beginning from random trees with 4 simultaneous independent Chains, performing 2 million replicates, sampling one tree every 100 generations [57]. The first 25% of the sampled trees were discarded as burn-in and a majority rule consensus tree of all remaining trees was calculated.

Branches that received bootstrap supports for MP, ML greater than or equal to 50% and Bayesian inference (BI) greater than or equal to 0.95 were considered as significantly supported. Phylogenetic trees were visualized using FigTree v1.4.2.

## 3. Results

### 3.1. Phylogenetic Analyses

**Figure 1:**
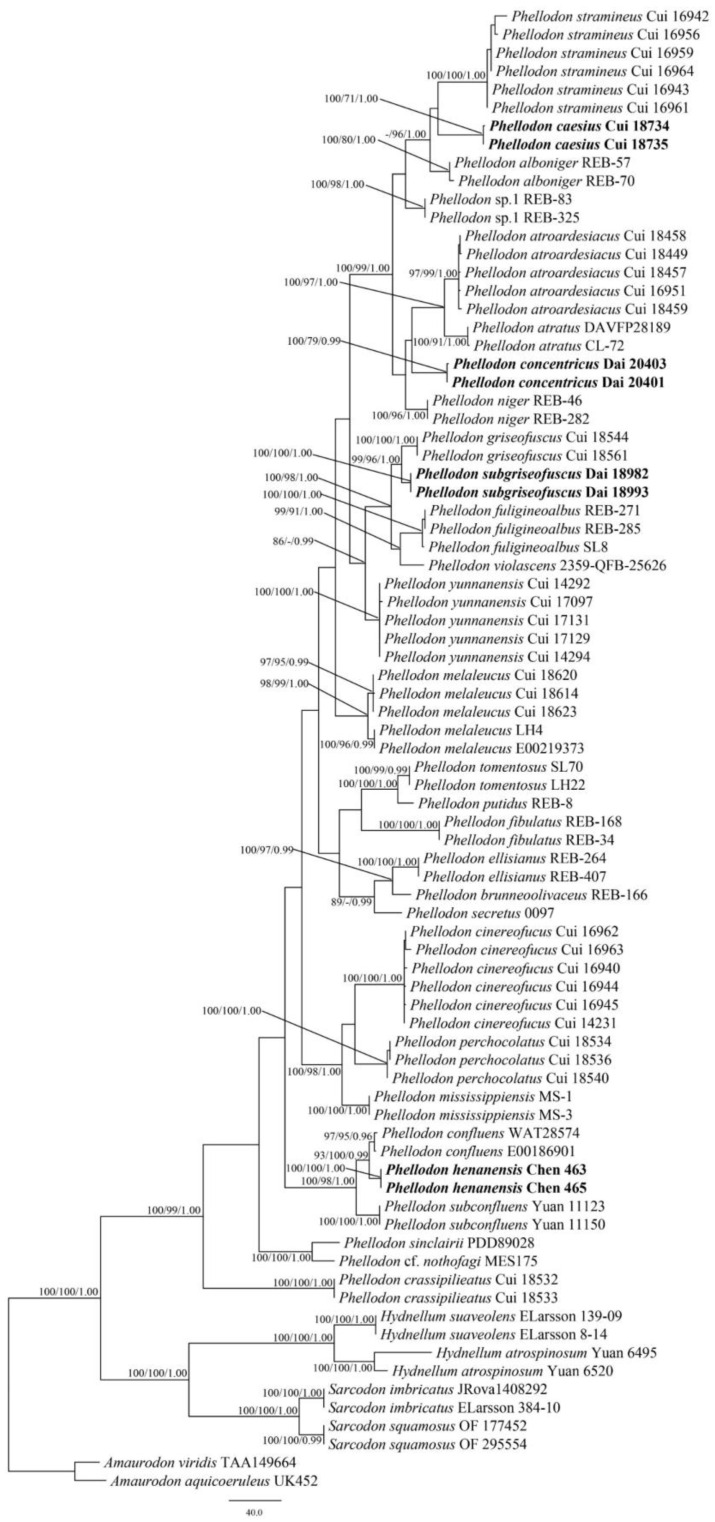
Maximum parsimony (MP) phylogram of the *Phellodon* species based on ITS + nLSU sequences data. The supported branches are labeled with parsimony bootstrap values higher than 50%, maximum likelihood bootstrap values higher than 50%, and Bayesian posterior probabilities more than 0.95. Bold names = New species.

The combined ITS + nLSU dataset included sequences from 81 fungal samples representing 35 taxa. The dataset had an aligned length of 2244 characters, including gaps (865 characters for ITS, 1379 characters for nLSU), of which 1564 characters were constant, 69 were variable and parsimony-uninformative, and 611 were parsimony-informative. Maximum parsimony analysis yielded 1205 equally parsimonious trees (TL = 1894, CI = 0.548, RI = 0.843, RC = 0.462, HI = 0.452). The best models for each region of the combined ITS + nLSU sequence dataset estimated and applied in the Bayesian analysis were both GTR + I + G models. Bayesian and ML analysis resulted in a topology similar to that from MP analysis. The Bayesian analysis resulted in a concordant topology with an average standard deviation of split frequencies = 0.005071. Only the MP tree is provided in Figure 1, and the MP (≥50%), ML (≥50%), and BI (≥0.95) are shown at the nodes.

**Figure 2:**
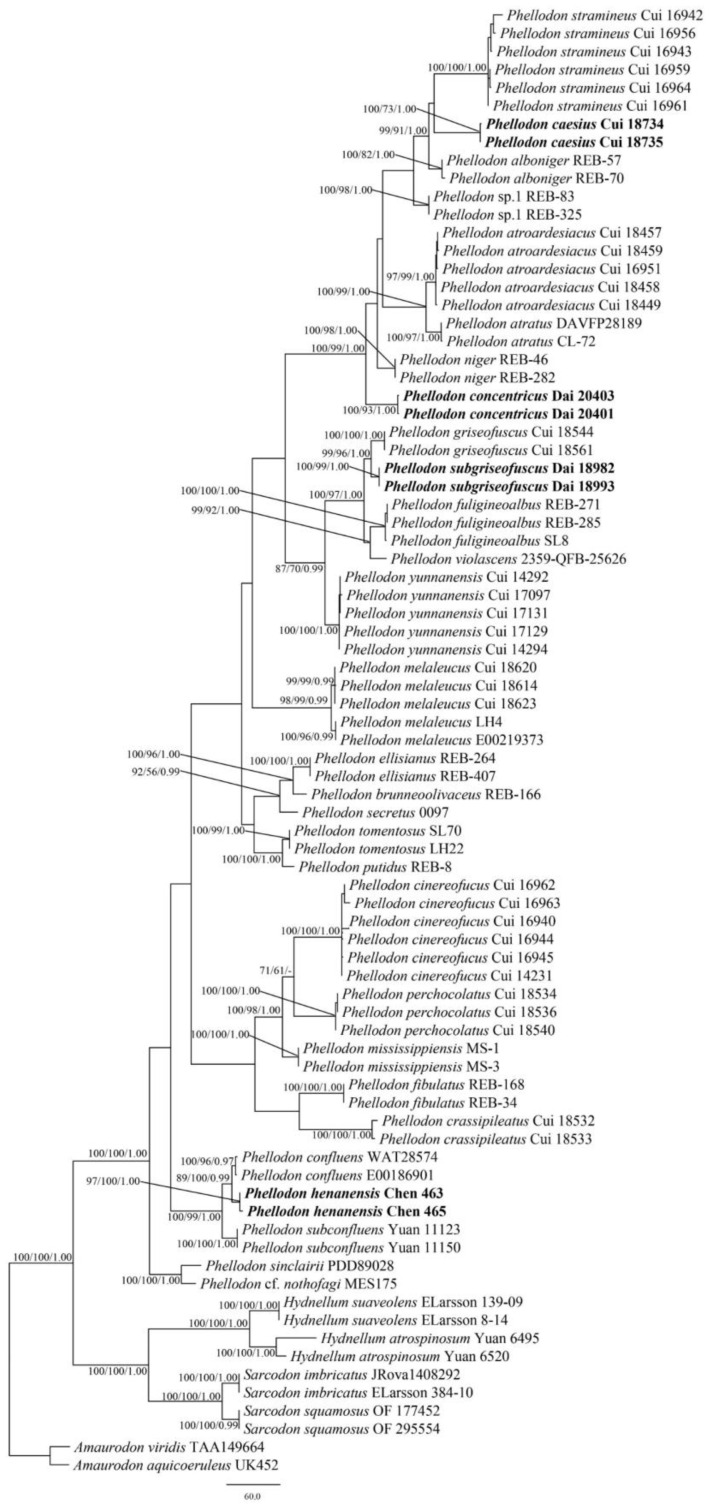
Maximum parsimony (MP) phylogram of the *Phellodon* species based on ITS + nLSU + nSSU + RPB1 + RPB2 sequences data. The supported branches are labeled with parsimony bootstrap values higher than 50%, maximum likelihood bootstrap values higher than 50%, and Bayesian posterior probabilities more than 0.95. Bold names = New species.

The combined 5-gene ITS + nLSU + nSSU + RPB1 + RPB2 dataset included sequences from 81 fungal samples representing 35 taxa. The dataset had an aligned length of 5597 characters, including gaps (865 characters for ITS, 1379 characters for nLSU, 1070 characters for nSSU, 1204 characters for RPB1, 1079 characters for RPB2), of which 4513 characters were constant, 199 were variable and parsimony-uninformative, and 885 were parsimony-informative. Maximum parsimony analysis yielded 2389 equally parsimonious trees (TL = 2389, CI = 0.615, RI = 0.857, RC = 0.527, HI = 0.385). The best-fit evolutionary models applied in Bayesian analyses were selected by jModelTest for each region of the five genes, the model for ITS, nLSU, nSSU, RPB1, and RPB2 was GTR + I+ G with an equal frequency of nucleotides. Bayesian and ML analysis resulted in a topology similar to that from MP analysis. The Bayesian analysis resulted in a concordant topology with an average standard deviation of split frequencies = 0.004330. Only the MP tree is provided in Figure 2, and the MP (≥50%), ML (≥50%), and BI (≥0.95) are shown at the nodes.

Both the ITS + nLSU dataset and the ITS + nLSU + nSSU + RPB1 + RPB2-based phylogenetic tree (Figure 1 and Figure 2) confirmed the affinities of the four new species within *Phellodon.* The four new species *P. caesius*, *P. concentricus*, *P. henanensis*, and *P. subgriseofuscus* formed distinct well-supported lineages distant from other species of *Phellodon*.

### 3.2. Taxonomy

**Figure 3:**
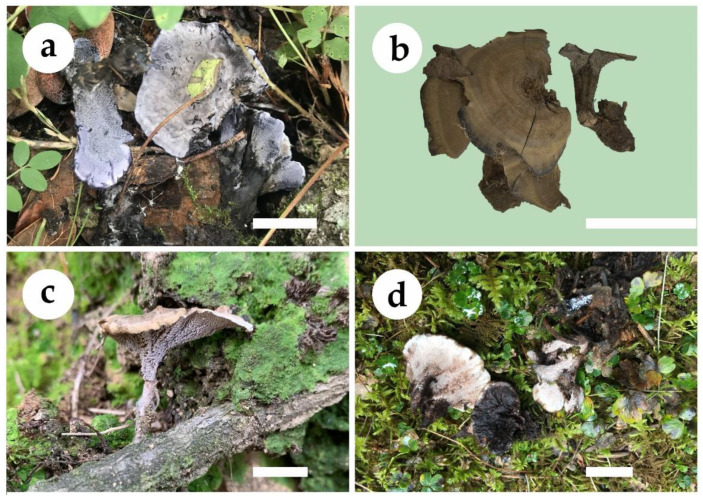
Basidiomata of *Phellodon* species. (**a**) *P. caesius*, (**b**) *P. concentricus*, (**c**) *P. henanensis*, and (**d**) *P. subgriseofuscus*. Scale bars: 2 cm.

**Figure 4:**
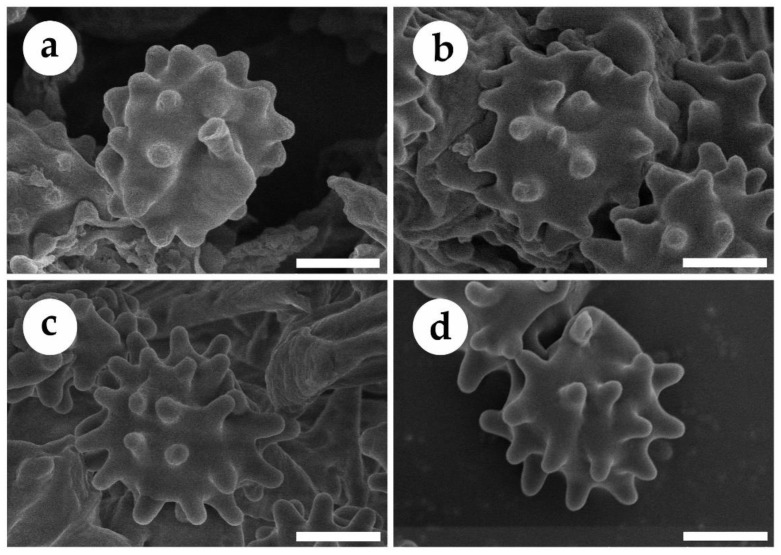
SEM of basidiospores of *Phellodon* species. (**a**) *P. caesius*, (**b**) *P. concentricus*, (**c**) *P. henanensis*, and (**d**) *P. subgriseofuscus*. Scale bars: 1.5 µm.

**Figure 5:**
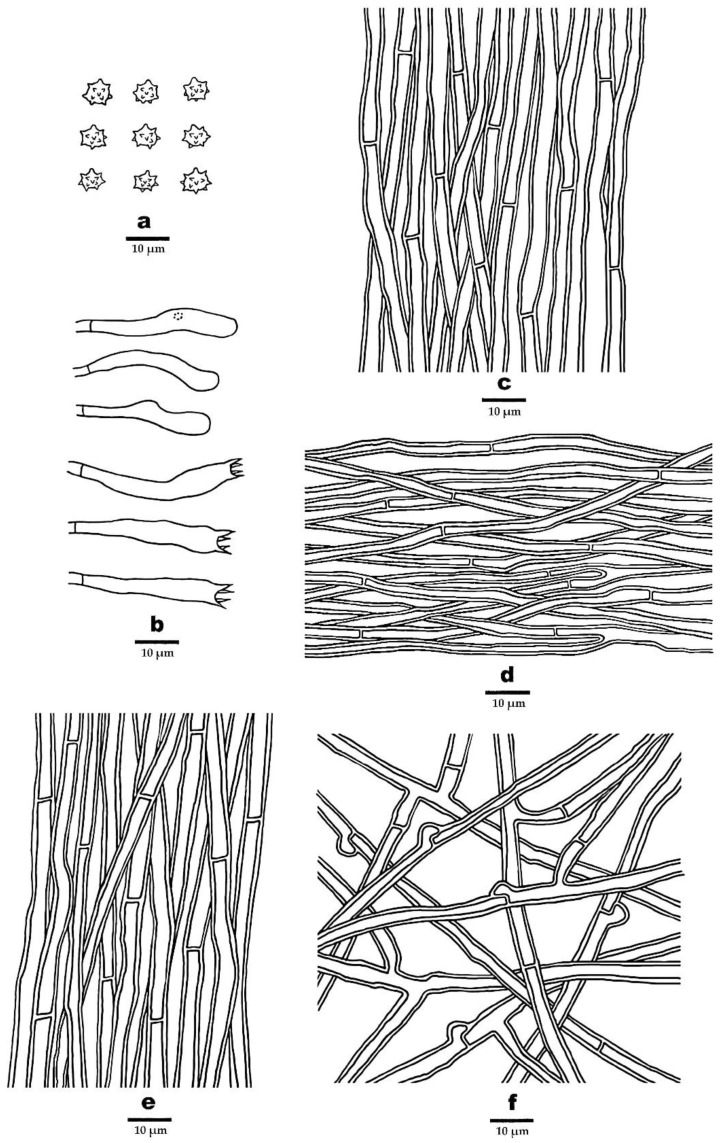
Microscopic structures of *P. caesius* (drawn from the holotype). (**a**) Basidiospores, (**b**) Basidia and basidioles, (**c**) Hyphae from context, (**d**) Hyphae from spines, (**e**) Hyphae from inner layer of stipe, and (**f**) Hyphae from surface layer of stipe.

***Phellodon caesius*** B.K. Cui & C.G. Song, sp. nov., Figure 3a, Figure 4a and Figure 5.

MycoBank: 846978

**Diagnosis**—Differs from other *Phellodon* species by its bluish-grey, dark grey to black grey pileus, ash grey to dark bluish-grey spines, and the presence of both simple septa and clamp connections on generative hyphae of the surface layer of stipe.

**Etymology**—*caesius* (Lat.), refers to the bluish-grey pileus.

**Holotype**—CHINA. Sichuan Province, Xiaojin County, on the ground of forest dominated by *Quercus aquifolioides*, alt. 3320 m, 3 September 2021, Cui 18734 (BJFC 045001).

**Fruitbody**—Basidiomata annual, centrally or eccentrically stipitate, single to concrescent, with a light fenugreek odor when dry. Pileus slightly convex in the middle, plicate, up to 3.6 cm in diam, and 0.7 cm thick at the center. Pileal surface bluish-grey, dark bluish-grey to black grey when fresh and becoming pale mouse grey to mouse grey upon drying, azonate, fibrillose to spongy; margin white to ash grey when fresh, and becoming pale mouse grey upon drying, up to 2 mm wide. Context tough, dark violet to dark grey upon drying, up to 3 mm thick. Spines soft, white, ash grey to dark bluish-grey when fresh, becoming fragile, pale mouse grey to ash grey upon drying, up to 2 mm long. Stipe cylindrical, glabrous, dark grey to black in outer layer, black in the inner layer, up to 2.2 cm long, 1.2 cm in diam. 

**Hyphal structure**—Hyphal system monomitic; generative hyphae in context, spines, and the inner layer of stipe with simple septa, generative hyphae in the surface layer of stipe mostly with simple septa, occasionally with clamp connections; all the hyphae IKI–, CB–; tissues turned olive green in KOH. Generative hyphae in context clay-buff, thick-walled, rarely branched, regularly arranged, 2.5–5 µm in diam. Generative hyphae in spines dark clay-buff, thick-walled, occasionally branched, regularly arranged, 2–3.5 µm in diam. Generative hyphae in the inner layer of stipe clay-buff to fuscous, thick-walled, rarely branched, regular arranged, 3–5 µm in diam; generative hyphae in the surface layer of stipe fuscous, thick-walled, branched, interwoven, 3–6 µm in diam. 

**Cystidia**—Cystidia and other sterile hyphal elements absent. 

**Basidia**—Clavate, bearing four sterigmata and a basal simple septum, 29–53 × 5.5–7 µm; sterigmata 2–5.5 µm long; basidioles similar to basidia in shape, but slightly smaller. 

**Spores**—Basidiospores subglobose to globose, hyaline, thin-walled, echinulate, IKI–, CB–, 4–5.6(–6) × (3.8–)4–5.2 µm, *L* = 4.87 µm, *W* = 4.48 µm, *Q* = 1–1.25 (*n* = 60/2, without the ornamentation). 

**Additional specimen (paratype) examined**—CHINA. Sichuan Province, Xiaojin County, on the ground of forest dominated by *Quercus aquifolioides*, alt. 3320 m, 3 September 2021, Cui 18735 (BJFC 045002).

**Ecological habits**—*P. caesius* was found on the ground of forest dominated by trees of *Quercus aquifolioides*, under a temperate climate at high altitude regions in Southwest China.

**Figure 6:**
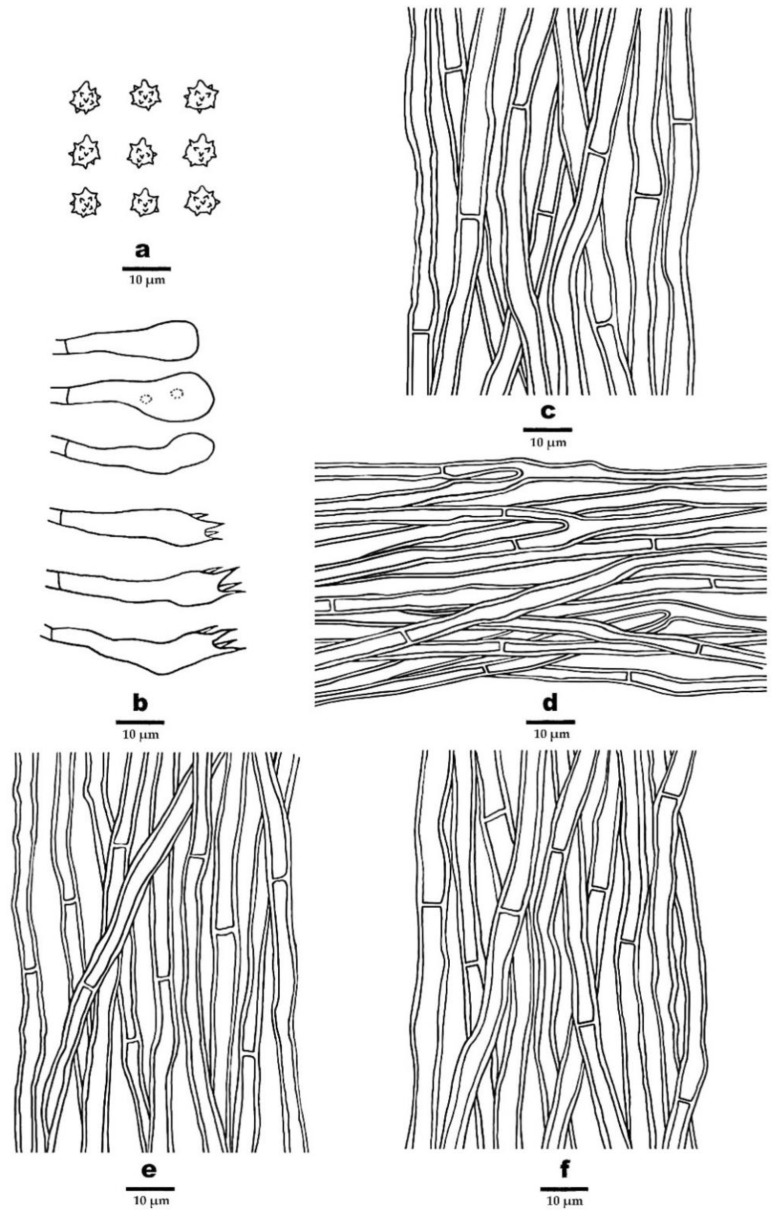
Microscopic structures of *P. concentricus* (drawn from the holotype). (**a**) Basidiospores, (**b**) Basidia and basidioles, (**c**) Hyphae from context, (**d**) Hyphae from spines, (**e**) Hyphae from inner layer of stipe, and (**f**) Hyphae from surface layer of stipe.

***Phellodon concentricus*** B.K. Cui and C.G. Song, sp. nov., Figure 3b, Figure 4b and Figure 6. 

MycoBank: 846979 

**Diagnosis**—Differs from other *Phellodon* species by its zonate pileal surface, dark grey context in pileus, and spongy basidiomata.

**Etymology**—*concentricus* (Lat.), refers to the concentric bands on pileal surface.

**Holotype**—CHINA. Yunnan Province, Yuxi County, Xinping, Mopanshan Forest Park, on the ground of forest dominated by *Quercus* sp., alt. 2088 m, 14 August 2019, Dai 20403 (BJFC 032071).

**Fruitbody**—Basidiomata annual, centrally or eccentrically stipitate, single to concrescent, with a strong fenugreek odor when dry. Pileus depressed, circular to irregular, up to 4.5 cm in diam, 0.3 cm thick at the center. Pileal surface deep olive to mouse grey upon drying, zonate, fibrillose to spongy at the center; margin fuscous to black upon drying, up to 5 mm wide. Context tough, dark grey upon drying, up to 1 mm thick. Spines soft when fresh, becoming fragile, ash grey upon drying, up to 2.5 mm long. Stipe cylindrical, spongy, deep olive, fuscous to black, up to 2.5 cm long, 1 cm in diam.

**Hyphal structure**—Hyphal system monomitic; generative hyphae with simple septa; all the hyphae IKI–, CB–; tissues turned light yellow-green to olive green in KOH. Generative hyphae in context dark yellowish-green, thick-walled, rarely branched, regularly arranged, 3–6.5 µm in diam. Generative hyphae in spines yellowish-brown to dark brown, slightly thick-walled, branched, regularly arranged, 2–4.5 µm in diam. Generative hyphae in stipe dark olive-green to black, thick-walled, rarely branched, regularly arranged, 2–6 µm in diam. 

**Cystidia**—Cystidia and other sterile hyphal elements absent.

**Basidia**—Clavate, bearing four sterigmata and a basal simple septum, 25–44 × 5.2–6.8 µm; sterigmata 3.5–6 µm long; basidioles similar to basidia in shape but slightly smaller.

**Spores**—Basidiospores subglobose to globose, hyaline, thin-walled, echinulate, IKI–, CB–, 5–6.2 × 4.5–5.5(–5.7) µm, *L* = 5.48 µm, *W* = 4.99 µm, *Q* = 1–1.22 (*n* = 60/2, without the ornamentation).

**Additional specimen (paratype) examined**—CHINA. Yunnan Province, Yuxi County, Xinping, Mopanshan Forest Park, on the ground of forest dominated by *Quercus* sp., alt. 2088 m, 14 August 2019, Dai 20401 (BJFC 032069).

**Ecological habits**—*P. concentricus* was found in forest dominated by trees of *Quercus* sp., under a subtropical climate.

**Figure 7:**
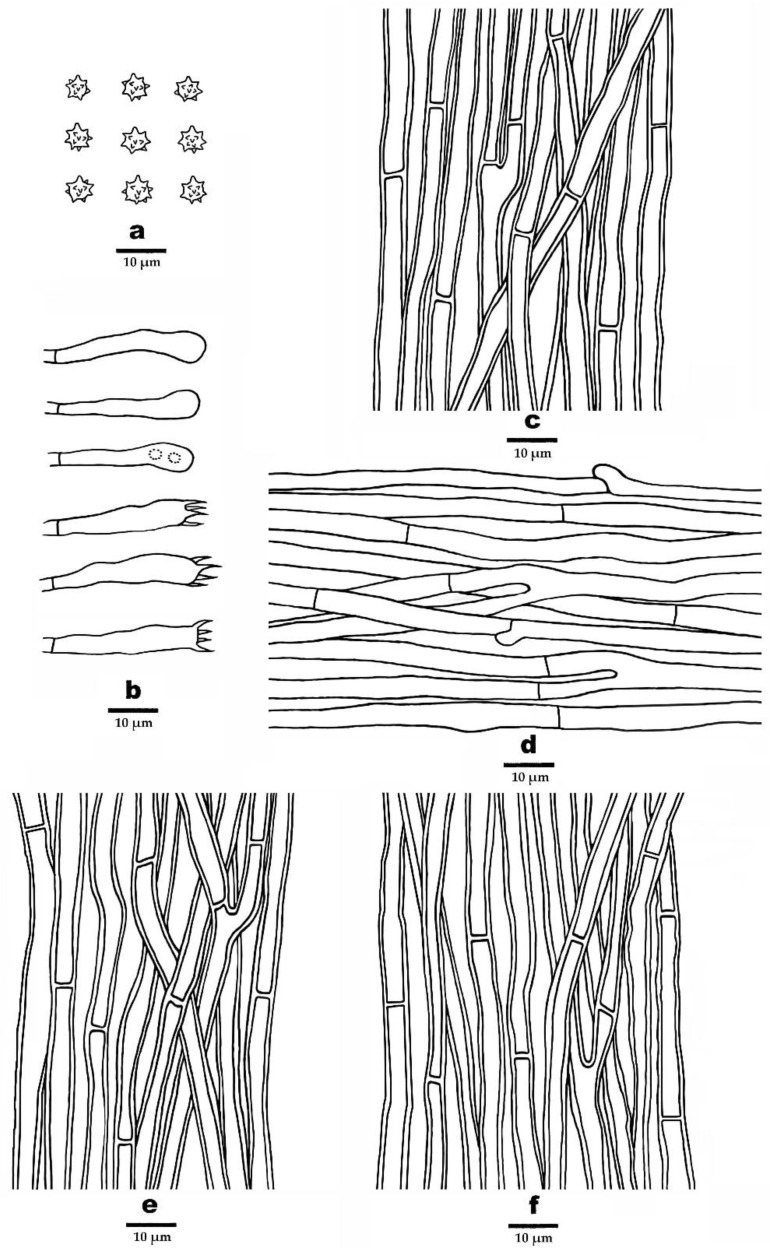
Microscopic structures of *P. henanensis* (drawn from the holotype). (**a**) Basidiospores, (**b**) Basidia and basidioles, (**c**) Hyphae from context, (**d**) Hyphae from spines, (**e**) Hyphae from inner layer of stipe, and (**f**) Hyphae from surface layer of stipe.

***Phellodon henanensis*** B.K. Cui and C.G. Song, sp. nov., Figure 3c, Figure 4c and Figure 7. 

MycoBank: 846980

**Diagnosis**—Differs from other *Phellodon* species by its ash grey, light vinaceous grey to light brown pileal surface, thin context in pileus, and the presence of both simple septa and clamp connections on generative hyphae of spines.

**Etymology**—*henanensis* (Lat.), refers to the holotype locality of the species in Henan Province.

**Holotype**—CHINA. Henan Province, Luanchuan County, Laojun Mountain, Jindian, on the ground of mixed forest, alt. 2000 m, 8 September 2020, Chen 463 (BJFC 045003). 

**Fruitbody**—Basidiomata annual, eccentrically stipitate, usually solitary, with a fenugreek odor when dry. Pileus depressed or shallow infundibuliform, up to 2.2 cm in diam, 0.3 cm thick at the center. Pileal surface ash grey, light vinaceous grey to light brown when fresh and becoming dark brown to black upon drying, azonate, fibrillose; margin cream to light brown when fresh, and becoming apricot-orange upon drying, up to 3 mm wide. Context tough, greyish-brown, up to 1 mm thick. Spines soft, ash grey to light brown when fresh, becoming fragile, vinaceous grey to greyish-brown upon drying, up to 1 mm long. Stipe cylindrical, glabrous, pale greyish-brown to pale mouse grey, up to 1.3 cm long, 0.2 cm in diam. 

**Hyphal structure**—Hyphal system monomitic; generative hyphae in context and stipe with simple septa, generative hyphae in spines mostly with simple septa, occasionally with clamp connections; all the hyphae IKI–, CB–; all tissues turned olive green in KOH. Generative hyphae in context hyaline to clay-buff, thick-walled, occasionally branched, regularly arranged, 2–6 µm in diam. Generative hyphae in spines hyaline to clay-buff, thin-walled, occasionally branched, regularly arranged, 2–4 µm in diam. Generative hyphae in stipe occasionally hyaline to dark brown, thick-walled, branched, regularly arranged, 2.5–5 µm in diam. 

**Cystidia**—Cystidia and other sterile hyphal elements absent. 

**Basidia**—Clavate, bearing four sterigmata and a basal simple septum, 24–46 × 4.5–5.5 µm; sterigmata 2–5 µm long; basidioles similar to basidia in shape, but slightly smaller. 

**Spores**—Basidiospores subglobose to globose, hyaline, thin-walled, echinulate, IKI–, CB–, (3.2–)3.8–5 × (3–)3.5–4.5(–4.8) µm, *L* = 4.17 µm, *W* = 3.84 µm, *Q* = 1–1.31 (*n* = 60/2, without the ornamentation). 

**Additional specimen (paratype) examined**—CHINA. Henan Province, Luanchuan County, Laojun Mountain, Jindian, on the ground of mixed forest, alt. 2000 m, 8 September 2020, Chen 465 (BJFC 045004).

**Ecological habits**—*P. henanensis* was found on the ground with a thin layer of moss, under a warm temperate continental monsoon climate.

**Figure 8:**
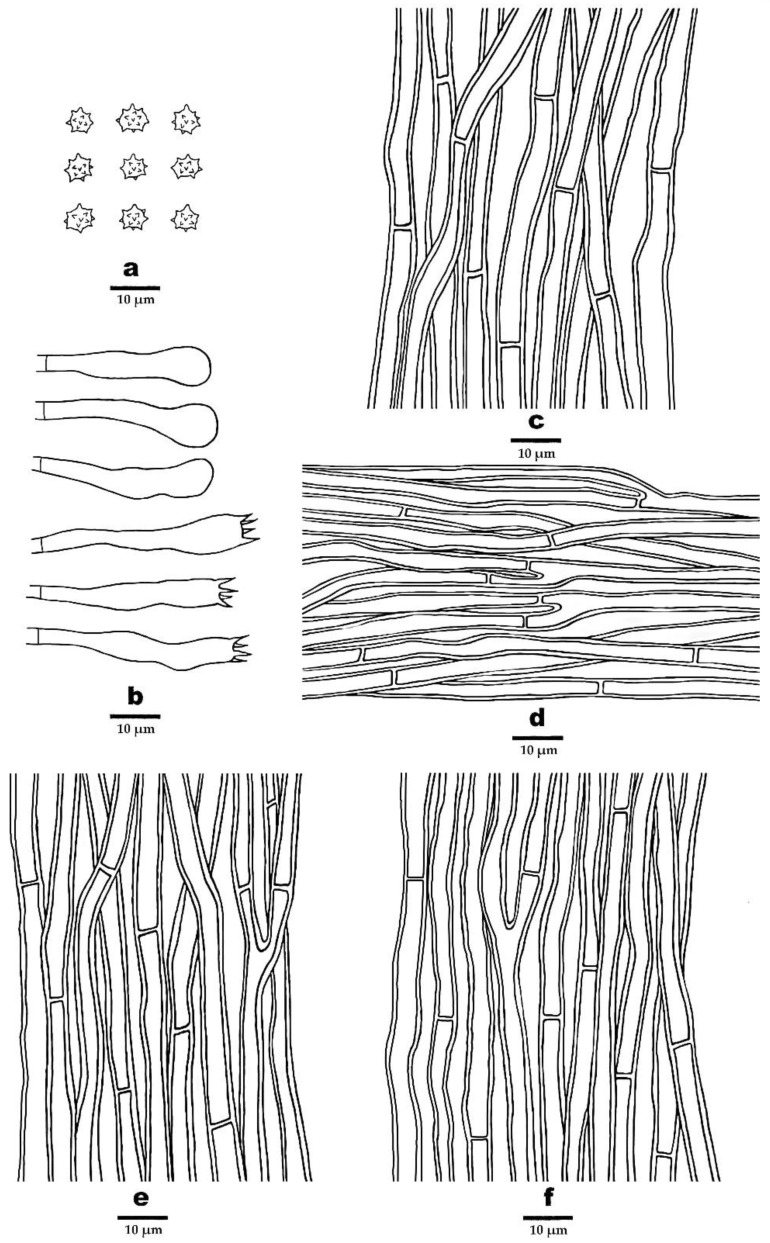
Microscopic structures of *P. subgriseofuscus* (drawn from the holotype). (**a**) Basidiospores, (**b**) Basidia and basidioles, (**c**) Hyphae from context, (**d**) Hyphae from spines, (**e**) Hyphae from inner layer of stipe, and (**f**) Hyphae from surface layer of stipe.

***Phellodon subgriseofuscus*** B.K. Cui and C.G. Song, sp. nov., Figure 3d, Figure 4d and Figure 8. 

MycoBank: 846981

**Diagnosis**—Differs from other *Phellodon* species by its fuscous to black pileal surface, white to light brown spines, and vinaceous grey context.

**Etymology**—*subgriseofuscus* (Lat.), refers to the new species resembling *P. griseofuscus* in morphology.

**Holotype**—CHINA. Gansu Province, Zhangye, Qilianshan Nature Reserve, Sidalong belay station, on the ground of forest dominated by *Picea crassifolia*, alt. 3000 m, 4 September 2018, Dai 18993 (BJFC 027462). 

**Fruitbody**—Basidiomata annual, eccentrically stipitate, single to concrescent, with a fenugreek odor when dry. Pileus circular to irregular, up to 4.8 cm in diam, 1.2 cm thick at the center. Pileal surface fuscous to black when fresh and becoming dark brown to fuscous upon drying, zonate, glabrous, with radially aligned stripes; margin white to dark brown when fresh, and becoming white to cream upon drying, up to 3 mm wide. Context tough, vinaceous grey upon drying, up to 3 mm thick. Spines soft, white to light brown when fresh, becoming fragile, cream to buff-yellow upon drying, up to 2.5 mm long. Stipe cylindrical, glabrous, greyish-brown, dark brown to fuscous, up to 3.3 cm long, 1.5 cm in diam. 

**Hyphal structure**—Hyphal system monomitic; generative hyphae with simple septa; all the hyphae IKI–, CB–; tissues turned olive green in KOH. Generative hyphae in context brown, thick-walled, rarely branched, regularly arranged, 2–6 µm in diam. Generative hyphae in spines hyaline to clay-buff, slightly thick-walled, branched, regularly arranged, 2–4 µm in diam. Generative hyphae in stipe hyaline to dark brown, thick-walled, occasionally branched, regularly arranged, 2–6 µm in diam. 

**Cystidia**—Cystidia and other sterile hyphal elements absent.

**Basidia**—Clavate, bearing four sterigmata and a basal simple septum, 27–43 × 5–7 µm; sterigmata 2–5.5 µm long; basidioles similar to basidia in shape, but slightly smaller. 

**Spores**—Basidiospores subglobose to globose, hyaline, thin-walled, echinulate, IKI–, CB–, 4–5 × (3–)3.2–4.8 µm, *L* = 4.47 µm, *W* = 3.9 µm, *Q* = 1–1.41 (*n* = 60/2, without the ornamentation). 

**Additional specimen (paratype) examined**—CHINA. Gansu Province, Zhangye County, Qilianshan Nature Reserve, Xishui belay station, on the ground of forest dominated by *Picea crassifolia*, alt. 2250 m, 3 September 2018, Dai 18982 (BJFC 027451).

**Ecological habits**—*P. subgriseofuscus* was found on the ground of forest dominated by trees of *Picea*, under a continental alpine sub-humid mountain climate. This species grows in well-watered bryophytes.

**Key to species of *Phellodon* from China**

1. Pileal surface straw buff-------------------------------------------------------------------------------*P. stramineus*
1. Pileal surface differently colored--------------------------------------------------------------------2
2. Pileal surface blackish-blue to dark grey or bluish-grey to dark bluish-grey-------------3
2. Pileal surface differently colored--------------------------------------------------------------------4
3. Clamp connections exist in spines------------------------------------------------------------------*P. atroardesiacus*
3. Clamp connections do not exist in spines---------------------------------------------------------*P. caesius*
4. Tissues color changed in KOH-----------------------------------------------------------------------5
4. Tissues color unchanged in KOH-------------------------------------------------------------------*P. subconfluens*
5. Pileal surface glabrous---------------------------------------------------------------------------------6
5. Pileal surface not glabrous----------------------------------------------------------------------------8
6. Pileal surface reddish-brown to cinnamon brown----------------------------------------------*P. cinereofuscus*
6. Pileal surface differently colored--------------------------------------------------------------------7
7. Pileal surface clay pink to brown-------------------------------------------------------------------*P. yunnanensis*
7. Pileal surface fuscous to black-----------------------------------------------------------------------*P. subgriseofuscus*
8. Clamp connections exist-------------------------------------------------------------------------------9
8. Clamp connections absent----------------------------------------------------------------------------*P. concentricus*
9. Pileal surface ash grey, light vinaceous grey to light brown---------------------------------*P. henanensis*
9. Pileal surface differently colored-------------------------------------------------------------------10
10. Clamp connections exist in spines----------------------------------------------------------------11
10. Clamp connections do not exist in spines-------------------------------------------------------*P. crassipileatus*
11. Spines brown after mature--------------------------------------------------------------------------*P. griseofuscus*
11. Spines white after mature---------------------------------------------------------------------------*P. perchocolatus*

## 4. Discussion

Based on the phylogenetic analyses, 29 species of *Phellodon* grouped together (Figure 1 and Figure 2), including four new species from China: *P. caesius*, *P. concentricus*, *P. henanensis*, and *P. subgriseofuscus*. Our phylogenetic results are consistent with previous observations [27,28], and further information on the phylogeny and taxonomy of *Phellodon* is supplied. During the investigations of *Phellodon*, information on distribution areas and ecological habits was also obtained (Table 2).

*Phellodon caesius* is clustered together with *P. alboniger* and *P. stramineus* in our phylogenetic trees (Figure 1 and Figure 2). Morphologically, *P. alboniger* is similar to *P. caesius* in having a spongy pileal surface, and white, brownish-grey to burnt umber spines. However, *P. alboniger* differs from *P. caesius* by its white to greyish-orange pileal surface, and longer spines (up to 2 mm [11]). *Phellodon stramineus* is similar to *P. caesius* in having solitary or gregarious basidiomata, ash-grey spines, and similar-sized basidiospores [27]. However, *P. stramineus* differs from *P. caesius* in its straw-buff pileal surface and the absence of clamp connections in stipe [27]. *Phellodon atroardesiacus* B.K. Cui and C.G. Song are morphologically similar to *P. caesius* in their blackish-blue to dark grey pileal surface and the dark greyish-blue to ash grey spines [27]. Surprisingly, they are not closely related as demonstrated in our phylogenetic analyses (Figure 1 and Figure 2). *Phellodon atroardesiacus* differs from the newly described *P. caesius* by its smaller basidiospore size (4–5 × (3–)3.5–4.5 in *P. atroardesiacus* vs. 4–5.6(–6) × (3.8–)4–5.2 in *P. caesius* [27]).

*Phellodon concentricus* is closely related to *P. niger* in our phylogenetic analyses (Figure 1 and Figure 2). Morphologically, *P. niger* is similar to *P. concentricus* in having single to concrescent basidiomata, crowded spines, and a spongy pileal surface. However, *P. niger* can be distinguished by its dark blue to black context, white, grey, or bluish-grey spines, and the smaller basidiospores size (5–6 × 4–5 μm in *P. niger* vs. 5–6.2 × 4.5–5.5(–5.7) µm in *P. concentricus* [11]).

*Phellodon henanensis* and *P. confluens* were clustered together and then grouped with *P. subconfluens* in our phylogenetic analyses (Figure 1 and Figure 2). *Phellodon confluens* is similar to *P. henanensis* in having shallow infundibuliform pileus, and the same-colored pileus. However, *P. confluens* differs from *P. henanensis* by its larger pileus measuring 3–10 cm, and the smaller basidiospores size (3.6–4.3 × 3.3–4 µm in *P. confluens* vs. (3.2–)3.8–5 × (3–)3.5–4.5(–4.8) µm in *P. henanensis* [18]). Morphologically, *P. subconfluens* is similar to *P. henanensis* in having a fenugreek odor when dry and short spines (up to 1 mm). However, *P. subconfluens* differs from *P. henanensis* by its greyish-buff, brownish-orange to reddish-brown pileal surface, cream to greyish-buff spines, and the smaller basidiospores size ((3.0–)3.1–4.1(–4.8) × (2.5–)2.9–3.5(–3.8) μm in *P. subconfluens* vs. (3.2–)3.8–5 × (3–)3.5–4.5(–4.8) µm in *P. henanensis* [29]).

*Phellodon subgriseofuscus* is closely related to *P. griseofuscus* B.K. Cui and C.G. Song in our phylogenetic analyses (Figure 1 and Figure 2). Morphologically, *P. griseofuscus* is similar to *P. subgriseofuscus* in having dark brown or black pileal surface and white to light brown spines. However, *P. griseofuscus* can be distinguised by its shorter spines (up to 1 µm), and clamp connections in generative hyphae of pileus and stipe [28].

The diversity and evolutionary relationships of *Phellodon* species can be objectively revealed by combining traditional morphological observation with molecular systematics methods. In the past, only a few numbers of publications had used phylogenetic analyses of the *Phellodon* genus, and the majority of those studies had only used the ITS sequences of a few species [11,24,25,29]. Song et al. [27,28] conducted phylogenetic analysis of *Phellodon* based on 5-gene sequences (ITS + nLSU + nSSU + RPB1 + RPB2), which undoubtedly filled in the blank of multiple gene fragments of *Phellodon*. In this study, both ITS + LSU and ITS + LSU + SSU + RPB1 +RPB2 datasets share a similar topology with Song et al. [27,28] but with discrepant bootstrap values.

*Phellodon* species frequently grow beneath pine needles or oak leaves, which serve to prevent water loss, in damp woodlands covered in dense mosses. Specimens collected in China were gathered from forests of pinaceae, fagaceae, or mixed trees (Table 2). It revealed that *Phellodon* species are host-biased, providing an additional foundation for species discovery and identification. The specimens were collected from northeast, southwest, northwest, and central China at elevations ranging from 870 to 3320 m, which indicated that the genus is a widespread species.

With the addition of the species discussed above, there are now 12 taxa in *Phellodon* known from China. The identification and descriptions of stipitate hydnoid fungi in this paper can enrich the species diversity of *Phellodon* and promote the taxonomy and phylogeny of the genus. The combination of morphological and phylogenetic methods will contribute to the exploration of species diversity. Additionally, it suggested that other *Phellodon* species might be discovered by combining the evidence of morphological characters, molecular data, and ecological habits. A fully resolved phylogeny for species in *Phellodon* requires evolutionary information from more samples.

## Figures and Tables

**Table 1:** A list of species, specimens, and GenBank accession numbers of sequences used in this study.

Species	Specimen No.	Locality	GenBank Accession No.				
			ITS	nrLSU	nuSSU	RPB1	RPB2
*Amaurodon aquicoeruleus*	UK 452	Australia	AM490944	AM490944	-	-	-
*A. viridis*	TAA 149664	Russia	AM490942	AM490942	-	-	-
*Hydnellum atrospinosum*	Yuan 6520	China	MW579912	-	MW579912	-	-
*H. atrospinosum*	Yuan 6495	China	MW579938	MW579885	MW579911	-	-
*H. suaveolens*	ELarsson 139-09	Norway	MK602734	MK602734	-	-	-
*H. suaveolens*	ELarsson 8-14	Sweden	MK602735	MK602735	-	-	-
*P. alboniger*	REB-70	USA	KC571749	-	-	-	-
*P. alboniger*	REB-57	USA	JN135206	-	-	-	-
*P. atratus*	CL-72	Canada	MK281471	-	-	-	-
*P. atratus*	DAVFP 28189	Canada	HQ650766	-	-	-	-
*P. atroardesiacus*	Cui 18449	China	MZ221189	MZ225598	MZ225636	-	-
*P. atroardesiacus*	Cui 18457	China	MZ225577	MZ225599	MZ225637	-	-
*P. atroardesiacus*	Cui 18458	China	MZ225633	MZ225600	MZ225638	-	-
*P. atroardesiacus*	Cui 18459	China	MZ225634	MZ225601	MZ225639	-	-
*P. atroardesiacus*	Cui 16951	China	MZ225632	MZ225597	MZ225635	MZ343209	MZ343197
*P. brunneoolivaceus*	REB-166	USA	KC571752	-	-	-	-
*P. caesius*	Cui 18734	China	**OP751005**	**OP751407**	**OP751414**	**OP755302**	**OP755305**
*P. caesius*	Cui 18735	China	**-**	**OP751408**	**OP751415**	**OP755303**	**-**
*P. cinereofuscus*	Cui 14231	China	MZ225579	-	-	-	-
*P. cinereofuscus*	Cui 16940	Australia	MZ225580	MZ225602	MZ225640	MZ343210	MZ343198
*P. cinereofuscus*	Cui 16944	China	MZ225581	MZ225603	MZ225641	MZ343211	MZ343199
*P. cinereofuscus*	Cui 16945	China	MZ225582	MZ225604	MZ225642	-	-
*P. cinereofuscus*	Cui 16962	China	MZ225583	MZ225605	MZ225643	MZ352084	MZ343200
*P. cinereofuscus*	Cui 16963	China	MZ225584	MZ225606	MZ225644	MZ352085	MZ343201
*P. concentricus*	Dai 20401	China	**-**	**OP751406**	**OP751413**	**OP755301**	**-**
*P. concentricus*	Dai 20403	China	**OP751004**	**OP751405**	**OP751412**	**-**	**-**
*P. confluens*	WAT 28574	UK	EU622361	-	-	-	-
*P. confluens*	E00 186901	UK	EU622362	-	-	-	-
*P. crassipilieatus*	Cui 18532	China	OL449267	OL439037	OL439027	-	-
*P. crassipilieatus*	Cui 18533	China	OL449268	OL439038	OL439028	-	-
*P. ellisianus*	REB-264	USA	KC571757	-	-	-	-
*P. ellisianus*	REB-407	USA	KC571759	-	-	-	-
*P. fibulatus*	REB-168	USA	JN135205	-	-	-	-
*P. fibulatus*	REB-34	USA	KC571761	-	-	-	-
*P. fuligineoalbus*	REB-271	USA	KC571760	-	-	-	-
*P. fuligineoalbus*	REB-285	USA	JN135196	-	-	-	-
*P. fuligineoalbus*	SL8	-	EU622316	-	-	-	-
*P. griseofuscus*	Cui 18544	China	OL449265	OL439035	OL439025	OL456229	OL449087
*P. griseofuscus*	Cui 18561	China	OL449266	OL439036	OL439026	-	-
*P. henanensis*	Chen 463	China	**OP751002**	**-**	**OP751410**	**-**	**-**
*P. henanensis*	Chen 465	China	**OP751003**	**OP751404**	**OP751411**	**-**	**-**
*P. melaleucus*	LH4	UK	EU622368	-	-	-	-
*P. melaleucus*	E00219373	UK	EU622369	-	-	-	-
*P. melaleucus*	Cui 18614	China	OL449262	OL439032	OL439022		-
*P. melaleucus*	Cui 18620	China	OL449263	OL439033	OL439023	-	-
*P. melaleucus*	Cui 18623	China	OL449264	OL439034	OL439024	-	-
*P. mississippiensis*	MS-1	USA	JN247563	-	-	-	-
*P. mississippiensis*	MS-3	USA	JN247564	-	-	-	-
*P. niger*	REB-46	USA	JN135202	-	-	-	-
*P. niger*	REB-282	USA	KC571766	-	-	-	-
*P.* cf. *nothofagi*	MES-175	Chile	MH930224	-	-	-	-
*P. perchocolatus*	Cui 18534	China	OL449259	OL439029	OL439020		-
*P. perchocolatus*	Cui 18536	China	OL449260	OL439030	-	-	-
*P. perchocolatus*	Cui 18540	China	OL449261	OL439031	OL439021	-	-
*P. putidus*	REB-8	USA	JN135200	-	-	-	-
*P. secretus*	0097	Russia	MG597404	-	-	-	-
*P. sinclairii*	PDD 89028	New Zealand	GU222291	-	-	-	-
*P. stramineus*	Cui 16942	China	MZ225585	MZ225607	MZ225645	MZ352086	-
*P. stramineus*	Cui 16943	China	MZ225586	MZ225608	MZ225646	MZ352087	MZ343202
*P. stramineus*	Cui 16956	China	MZ225587	MZ225609	MZ225647	MZ352088	MZ343203
*P. stramineus*	Cui 16959	China	MZ225588	MZ225610	MZ225648	MZ352089	MZ343204
*P. stramineus*	Cui 16961	China	MZ225589	MZ225611	MZ225649	MZ352090	MZ343205
*P. stramineus*	Cui 16964	China	MZ225590	MZ225612	MZ225650	MZ352091	-
*P. subconfluens*	Yuan 11123	China	MK677464	-	-	-	-
*P. subconfluens*	Yuan 11150	China	MK677465	-	-	-	-
*P. subgriseofuscus*	Dai 18982	China	**OP751000**	**-**	**-**	**-**	**-**
*P. subgriseofuscus*	Dai 18993	China	**OP751001**	**OP751403**	**OP751409**	**-**	**OP755301**
*Phellodon* sp.1	REB-83	USA	KC571747	-	-	-	-
*Phellodon* sp.1	REB-325	USA	KC571748	-	-	-	-
*P. tomentosus*	SL70	UK	EU622381	-	-	-	-
*P. tomentosus*	LH22	UK	EU622382		-	-	-
*P. yunnanensis*	Cui 14292	China	MZ225591	-	-	-	-
*P. yunnanensis*	Cui 14294	China	MZ225592	-	-	-	-
*P. yunnanensis*	Cui 17097	China	MZ225593	MZ225613	MZ225651	-	MZ343206
*P. yunnanensis*	Cui 17129	China	MZ225594	MZ225614	MZ225652	-	MZ343207
*P. yunnanensis*	Cui 17131	China	MZ225595	MZ225615	MZ225653	-	MZ343208
*P. violascens*	2359-QFB-25626	-	KM406977	-	-	-	-
*Sarcodon imbricatus*	JRova 1408292	Sweden	MK602746	MK602746	-	-	-
*S. imbricatus*	ELarsson 384-10	Norway	MK602747	MK602747	-	-	-
*S. squamosus*	OF 177452	Norway	MK602768	MK602768	-	-	-
*S. squamosus*	OF 295554	Norway	MK602769	MK602769	-	-	-

New sequences are shown in bold.

**Table 2:** The main morphological characteristics of species in *Phellodon* described in China.

Species	Distribution in China	Ecological Habits	Alt.	Pileal Surface	Spines Color	Spines Size (mm)	Clamp Connection	Basidios-Pores (µm)	References
*P. atroardesiacus*	Xizang Autonomous Region	in *Pinus densata* forest	2900 m	blackish-blue to dark grey when fresh	dark greyish-blue to ash grey when fresh	up to 5	occasionally with clamp connections in spines	4–5 × (3–) 3.5–4.5	Song et al., 2021 [27]
*P. caesius*	Sichuan Province	on the ground of forest dominated by *Quercus aquifolioides*	3320 m	bluish-grey, dark bluish-grey to black-grey when fresh	white, ash grey to dark bluish-grey when fresh	up to 2	occasionally with clamp connections in the surface layer of stipe	4–5.6(–6) × (3.8–)4–5.2	This study
*P. cinereofuscus*	Yunnan Province	on the ground of forest dominated by *Pinus* and Fagaceae forest, and mixed forest	1800–2250 m	reddish-brown to cinnamon brown when fresh	greyish-brown to white when fresh	up to 6	unclamped	4–5 × (3.5–) 4–4.5	Song et al., 2021 [27]
*P. concentricus*	Yunnan Province	on the ground of forest dominated by *Quercus*	2088 m	dark olive to mouse grey when dry	ash grey when dry	up to 2.5	unclamped	5–6.2 × 4.5–5.5(–5.7)	This study
*P. crassipileatus*	Sichuan Province	on the ground of forest dominated by *Quercus*	1190 m	pale brown to dark brown when fresh	white when fresh	up to 3	with clamp connections in pileus and stipe	(3.5–) 4–5 × 4–5	Song et al., 2022 [28]
*P. griseofuscus*	Sichuan Province	i on the ground of forest dominated by *Pinus* and *Picea*	2400 m	dark brown to black when fresh	white when young and brown with age when fresh	up to 1	with clamp connections in spines	4–5 × 3.5–4.5	Song et al., 2022 [28]
*P. henanensis*	Henan Province	on the ground of mixed forest	2000 m	ash grey, light vinaceous grey to light brown when fresh	ash grey to light brown when fresh	up to 1	occasionally with clamp connections in spines	(3.2–)3.8–5 × (3–)3.5–4.5(–4.8)	This study
*P. perchocolatus*	Sichuan Province	on the ground of forest dominated by *Quercus*	1190 m	brown to greyish-brown when fresh	white when fresh	up to 3	with clamp connections in spines	4–5 (–5.5) × (3.5–) 4–4.5 (–5)	Song et al., 2022 [28]
*P. stramineus*	Yunnan Province	on the ground of forest dominated by *Pinus yunnanensis* and Fagaceae	2250 m	straw buff when fresh	dark grey to ash grey when fresh	up to 3	unclamped	4–5.5 (–6) × 4–5 (–5.5)	Song et al., 2021 [27]
*P. subconfluens*	Liaojing Province	on the ground of forest dominated by *Quercus*	870 m	greyish-buff, brownish-orange to reddish-brown when fresh	cream to greyish-buff when fresh	up to 1	unclamped	(3.0–) 3.1–4.1 (–4.8) × (2.5–) 2.9–3.5 (–3.8)	Mu et al., 2019 [29]
*P. subgriseofuscus*	Gansu Province	on the ground of forest dominated by *Picea crassifolia*	2250–3000 m	dark brown to black when fresh	white to light brown when fresh	up to 2.5	unclamped	4–5 × (3–)3.2–4.8	This study
*P. yunnanensis*	Yunnan Province	on the ground of *Pinus* and Fagaceae forest or *Pinus* forest or *Pinus armandii* and *Rhododendron* forest	2300–2600 m	clay pink to brown when fresh	pale brown to white when fresh	up to 5	occasionally with clamp connections in stipe	3.5–4.5(–5) × 3–4 (–4.5)	Song et al., 2021 [27]

## Data Availability

The data and results of this study are available upon reasonable request. Please contact the main author of this publication.
